# Prognostic Factors of AL-PCMM and AL-MM: A Single-Center Retrospective Study

**DOI:** 10.7150/ijms.61712

**Published:** 2022-03-14

**Authors:** Junhui Xu, Zhixiang Qiu, Miao Yan, Bingjie Wang, Zhengyang Song, Huihui Liu, Mangju Wang, Xinan Cen

**Affiliations:** 1 Department of Hematology, Peking University First Hospital, Beijing, China; 2 Department of Cardiology, Cardiovascular Hospital of Xiamen University, School of Medicine, Xiamen University, Xiamen, China

**Keywords:** AL amyloidosis, AL-PCMM, AL-MM, prognostic factors, BU staging system

## Abstract

**Background:** Patients with amyloid light-chain (AL) amyloidosis with a bone marrow plasma cell ratio > 10% (AL-PCMM) have a poorer prognosis than patients with AL amyloidosis with a bone marrow plasma cell ratio of <10% (AL-only), similar to that of patients with AL amyloidosis and multiple myeloma (AL-MM). However, the prognostic factors for AL-PCMM and AL-MM have not been studied.

**Methods:** A total of 49 patients with AL-PCMM or AL-MM in the Peking University First Hospital registry in 2010-2018 were enrolled. Clinical and follow-up data were collected. The relationship between clinical parameters and survival time was also assessed.

**Results:** Compared with patients with AL-PCMM, patients with AL-MM only had a higher incidence of bone marrow plasma cell ratio ≥ 20%. In AL-PCMM and AL-MM, the survival time was significantly shorter in patients with alkaline phosphatase (ALP) ≥ 187.5 IU/L, γ-glutamyl transpeptidase (GGT) ≥ 85 IU/L, total bilirubin (TBIL) ≥ 20 µmol/L, cardiac troponin I (CTNI) ≥ 0.1 ng/mL, ejection fraction (EF) < 50%, initial therapeutic effect (ITE) < very good partial response (VGPR), and Boston University (BU) staging system stage ≥ III. ALP at diagnosis was correlated with brain natriuretic peptide (BNP) level, CTNI level, and EF rather than TBIL level. Cox regression analyses revealed that BU staging system stage ≥ III (*P*=0.001, hazard ratio [HR]=5.579), ALP ≥ 187.5 IU/L (*P*=0.011, HR=3.563), and ITE < VGPR (*P*=0.002, HR=7.462) were independent significant risk factors for a poor prognosis of AL-PCMM and AL-MM.

**Conclusion:** ALP level, which is related to cardiac amyloidosis rather than liver involvement, can be a prognostic factor for this group of patients. A BU staging system stage ≥ III, ALP ≥ 187.5 IU/L, and ITE < VGPR were independent significant risk factors for a poor prognosis of AL-PCMM and AL-MM.

## Introduction

Primary amyloid light-chain (AL) amyloidosis is a systemic plasma cell disease with a poor prognosis that is related to the number and degree of involvement of affected organs. In 2004, Dispenzieri et al. collected and analyzed the clinical data of 242 AL amyloidosis patients from the Mayo Clinic and found that an *N*-terminal pro B-type natriuretic peptide (NT-proBNP) > 332 ng/L, cardiac troponin T (CTNT) > 0.035 μg/L, or cardiac troponin Ⅰ (CTNI) > 0.1 μg/L level was a poor prognostic factor 1. In 2012, Kumar et al. found that the difference between involved and uninvolved free light chains (dFLC) ≥ 180 mg/L was also an important prognostic factor 2. Therefore, a staging system combining CTNI, NT-proBNP, and dFLC was proposed. The median overall survival (OS) times for patients with stages I-IV disease were 94.1, 40.3, 14.0, and 5.8 months (*P*<0.001), respectively. In clinical practice, we often use the 2004 and 2012 Mayo Clinical AL Amyloidosis Staging System to stratify patients with AL amyloidosis. However, many centers are not capable of detecting NT-proBNP levels.

In 2019, the Boston University School of Medicine and Boston Medical Center proposed the Boston University (BU) staging system, which had the same effectiveness as the 2004 Mayo Clinical AL amyloidosis staging system for predicting the survival time of AL amyloidosis 3. In addition, Mollee et al. provided external validation of the BU staging system for AL amyloidosis, and only one patient was miscategorized according to the 2004 Mayo Clinical AL amyloidosis staging system 4. In addition to these prognostic staging systems, some studies also found that a bone marrow plasma cell (BMPC) ratio > 10%, M protein > 1 g/24 h, total bilirubin (TBIL), alkaline phosphatase (ALP), red blood cell distribution width, reactive vasodilation, D-dimer, preserved left ventricular ejection fraction, immunoparesis, and cytogenetic abnormalities such as T (11;14) are poor prognostic factors for AL amyloidosis 5-15.

There is consensus that patients with AL amyloidosis and concomitant hypercalcemia, renal failure, anemia, and lytic bone lesions attributable to clonal expansion of plasma cells (CRAB criteria) also have multiple myeloma. Patients with AL amyloidosis and a bone marrow plasma cell ratio > 10% (AL-PCMM) and AL amyloidosis with multiple myeloma (AL-MM) have a poorer prognosis than patients with AL amyloidosis with a BMPC ratio < 10% (AL-only) 16. However, the prognostic factors for AL-PCMM and AL-MM have not yet been studied. Reliable prognostic indicators for this group of patients are currently lacking. This study aimed to bridge this knowledge gap by collecting and analyzing clinical information and follow-up data to determine the prognostic factors of AL-PCMM and AL-MM.

## Methods

### Patients and institutional review board approval

Between January 1, 2010 and December 31, 2018, 52 patients with AL-PCMM or AL-MM were registered at Peking University First Hospital. This tertiary first-class hospital treats approximately 30 patients with AL amyloidosis each year. All patient data were anonymized. We searched the hospital medical records system using the disease codes to include all patients who met the enrollment criteria. Finally, only 49 patients were included in this study. One patient with incomplete data and two patients with a diagnosis interval of AL amyloidosis and MM of more than one month were excluded. Light and polarized microscopy for the presence of Congo red deposits and green birefringence in the biopsies of the affected parts were used to confirm the AL amyloidosis.

Organ involvement in AL amyloidosis was assessed according to the consensus criteria reported in the 10^th^ International Symposium on Amyloid and Amyloidosis 17. AL-MM was defined as AL amyloidosis with CRAB (serum Ca > 2.75 mmol/L, serum creatinine > 177 µmol/L, hemoglobin value > 20 g/L below the lower limit of normal, and one or more osteolytic lesions on skeletal radiography, computed tomography (CT), or positron emission tomography/CT (PET/CT). AL-PCMM was defined as AL amyloidosis with a BMPC ratio > 10% but without CRAB. Written informed consent was obtained from all participants. The Ethics Committee of Peking University First Hospital approved this study (no. 2017[1304]). This study was conducted in accordance with the principles of the Declaration of Helsinki.

### Data collection

Patient clinical information was obtained by hospital record review. The following clinical information at the time of diagnosis was extracted retrospectively: Ca, hemoglobin (Hb), 24-h urinary protein quantity (24-h UTP), albumin (Alb), serum creatinine (Scr), total bilirubin (TBIL), γ-glutamyl transpeptidase (GGT), alkaline phosphatase (ALP), cardiac troponin I (CTNI), brain natriuretic peptide (BNP), ejection fraction (EF), lactate dehydrogenase (LDH), and β-2 microglobulin (β2-MG). Bone destruction was determined using radiography, CT, magnetic resonance imaging (MRI), or PET/CT. A bone puncture was performed to determine the bone marrow plasma cell ratio. Based on the above-mentioned information, the best BU staging system for AL amyloidosis was determined.

### Treatment, initial therapeutic effect, and follow-up

At the time of diagnosis, the treatment regimens were divided into two drug combination regimens, melphalan and prednisone (MP), bortezomib and dexamethasone (BD), and lenalidomide and dexamethasone (RD), and a three-drug combination regimen, including melphalan plus prednisone and thalidomide (MPT), bortezomib plus cyclophosphamide and dexamethasone (BCD), bortezomib plus thalidomide and dexamethasone (BTD), bortezomib plus doxorubicin and dexamethasone (PAD), and bortezomib plus lenalidomide and dexamethasone (RVD). The 2016 International Myeloma Working Group consensus criteria were used to evaluate the response of multiple myeloma after the fourth course of chemotherapy 18, including progressive disease, stable disease, minimal response, partial response (PR), very good partial response (VGPR), complete response (CR), and stringent complete response (sCR).

A better initial therapeutic effect was defined as better than PR in terms of sCR, CR, and VGPR. Telephone interviews and retrospective review of hospital files were used to obtain patient survival data. The inability to communicate with the patient on the phone, which resulted in loss of survival data, was considered lost to follow-up. OS was defined as the time from diagnosis to the time of death (regardless of cause) or the last documented contact with the patient. AL amyloidosis-related death included renal failure caused by renal amyloidosis, cardiac amyloidosis (heart failure and arrhythmia), and digestive tract hemorrhage caused by digestive tract involvement.

### Statistical analyses

All analyses were performed using SPSS software (version 20.0; SPSS Institute). Categorical variables are reported as number and percentage. Chi-square and Fisher's exact tests were used to compare categorical variables among the different groups of patients. OS curves were analyzed using the Kaplan-Meier method and compared using the log-rank test for univariate analysis. Factors with values of *P*<0.10 on univariate analyses or acknowledged as clinically meaningful were included in the multivariate analysis. Multivariate analyses were performed using a Cox proportional hazards model. The Kolmogorov-Smirnov test was used to detect whether the values were normally distributed. Correlation analyses were performed using Spearman's correlation test. All quoted P values were obtained from two-sided tests. Statistical significance was set at *P*<0.05.

## Results

### Clinical characteristics and laboratory examination of AL-PCMM and AL-MM

A total of 49 patients with AL-PCMM or AL-MM were registered between January 1, 2010 and December 31, 2018. It is worth noting that 28/49 (57.1%) patients had PCMM and 21/49 (42.9%) had MM. Clinical information and laboratory examination results of all patients at the time of diagnosis are shown in Table 1. Patients with AL-MM had the same incidence of Alb < 25 g/L, 24-h UTP > 3.5 g, ALP ≥ 187.5IU/L, GGT ≥ 85 IU/L, TBIL ≥ 20 µmol/L, BNP ≥ 1500 pg/mL, EF < 50%, and BU staging system stage ≥ III, which represented AL amyloidosis severity. For tumor burden indicators, the incidence of LDH ≥ 240 IU/L and β2-MG > 3.5 mmol/L were the same for AL-PCMM and AL-MM, while AL-MM had a higher incidence of a BMPC ratio ≥ 20%. In addition, 7/28 (25.0%) patients with AL-MM had Hb levels of < 85 g/L. A total of 19/28 (67.8%) patients with AL-MM had bone destruction > 3; 3/28 (10.7%) patients with AL-MM had hypercalcemia, and 11/28 (39.2%) patients with AL-MM had renal dysfunction.

### Treatment and initial therapeutic effect of AL-PCMM and AL-MM

The treatment strategies are summarized in Table 2. At the time of diagnosis, 37 of the 49 patients were treated with a two-drug combination regimen, including 33 with BD, 3 with MP, and 1 with RD. Furthermore, 7 of 49 patients were treated with a three-drug combination regimen, including 2 with PAD, 2 with RVD, 1 with MTP, 1 with BCD, and 1 with BTD. In AL-PCMM, 16 patients were treated with a two-drug combination regimen, including 13 with BD, 2 with MP, and 1 with RD. In addition, 3 patients were treated with a three-drug combination regimen, including 1 with PAD, 1 with RVD, and 1 with BCD. In the AL-MM group, 21 patients were treated with a two-drug combination regimen, including 20 with BD and 1 with MP. In addition, 4 patients were treated with a three-drug combination regimen: 1 with PAD, 1 with RVD, 1 with MPT, and 1 with BCD. Finally, 5 patients received no treatment, including 2 with AL-PCMM and 3 with AL-MM. Among the two-drug and three-drug combination regimens, bortezomib-based regimens accounted for 89.1% and 85.7%, respectively. Only 4 (10.8%) patients receiving two-drug combination regimens and 1 (14.2%) patient receiving three-drug combination regimens underwent autologous stem cell transplantation during the course of the disease. There was no statistical difference in the proportion of patients with AL-MM with an ITE < VGPR (57.1% vs. 66.6%,* P*=0.564; Table 1).

### Organ involvement, death rate, and causes of death in patients with AL-PCMM and AL-MM

Organ involvement among the 49 patients with AL-PCMM or AL-MM was as follows: kidney (36 [73.4%]), liver (13 [26.5%]), heart (27 [55.1%]), skin (8 [16.3%]), gastrointestinal tract (6 [12.2%]), nerve (4 [8.2%]), soft tissue (4 [8.2%]), and pulmonary (1 [2%]). Fifteen (30.6%) patients had ≥ 3 involved organs, while 34 patients (69.4%) had 1 or 2 involved organs. There was no statistically significant difference in the incidence of the involvement of various organs and the number of organs involved between patients with AL-MM and AL-PCMM, especially the most commonly affected organs (kidneys, 71.4% vs. 76.1%; heart, 60.7% vs. 47.6%; liver, 25.0% vs. 28.5%) (Table S1).

A total of 31 of the 49 (63.3%) patients with AL amyloidosis died during the study period, including 19 of 34 patients (55.8%) with 1 or 2 involved organs and 12 of 15 patients (80%) with ≥3 involved organs. Specifically, 19 of 26 (73.0%) patients with heart involvement died. All 13 patients with liver involvement died, 6 of whom died of cardiac amyloidosis. A total of 24 of 36 patients (66.7%) with kidney involvement died, 12 of whom died of cardiac amyloidosis. Five of 6 patients (83.3%) with gastrointestinal tract involvement died, 3 of whom died of cardiac amyloidosis. Causes of death are listed in Table S2. Twelve (38.6%) patients did not have clear causes of death; among the remaining 19 patients, 13 (41.9%) died of cardiac amyloidosis, making it the leading cause of death. Other causes included renal failure caused by renal amyloidosis (2 [6.5%]), digestive tract hemorrhage caused by digestive tract involvement (2 [6.5%]), and other causes such as infection and hypercalcemia (2 [6.5%]).

### Survival analysis of AL-PCMM and AL-MM

**Figure 1:**
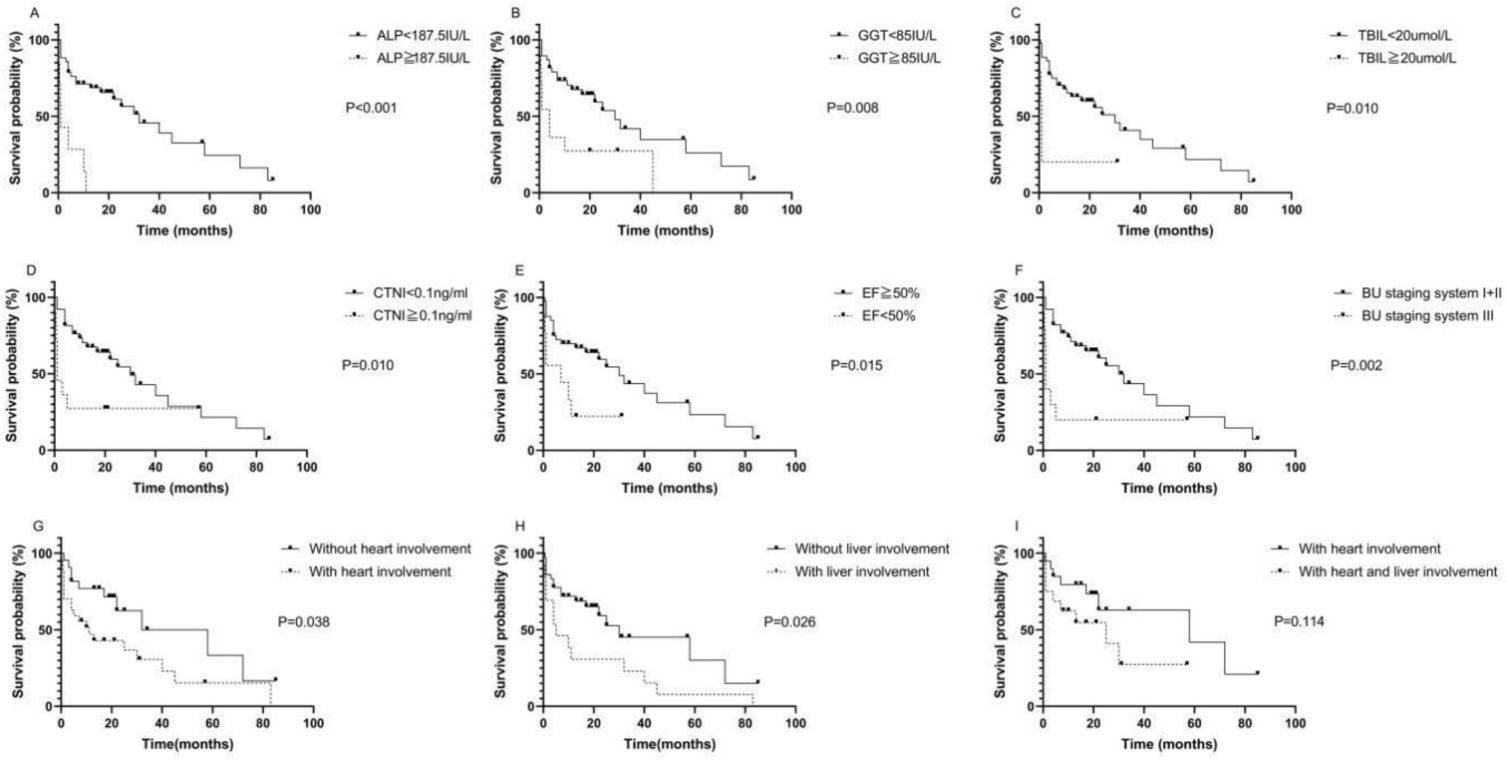
** Kaplan-Meier survival curves of AL-PCMM and AL-MM. A.** Survival difference between patients with an ALP < 187.5 IU/L and those with an ALP ≥ 187.5 IU/L (*P*<0.001). **B.** Survival difference between patients with a GGT < 85 IU/L and those with a GGT ≥ 85 IU/L (*P=*0.008). **C.** Survival difference between patients with a TBIL< 20 µmol/L and those with a TBIL ≥ 20 µmol/L (*P*=0.010). **D.** Survival difference between patients with a CTNI < 0.1 ng/mL and those with a CTNI ≥ 0.1 ng/mL (*P*=0.010). **E.** Survival difference between patients with an ejection fraction < 50% and those with an ejection fraction ≥ 50% (*P=*0.015). **F.** Survival difference between patients with BU staging system stages I + II and those with BU staging system stage III (*P=*0.002). **G.** Survival difference between patients with heart and those without heart involvement (*P*=0.038). **H.** Survival difference between patients with liver involvement and those without liver involvement (*P*=0.025)*.* I. Survival difference between patients with both heart and liver involvement and those with only heart involvement (*P=*0.114). AL-MM, amyloid light-chain amyloidosis and multiple myeloma; AL-PCMM, amyloid light-chain amyloidosis with a bone marrow plasma cell ratio > 10%; ALP, alkaline phosphatase; BU, Boston University; CTNI, cardiac troponin I; GGT, γ-glutamyl transpeptidase; TBIL, total bilirubin

The median survival time of the 49 patients was 32.26 months. The median follow-up time was 41.96 months. Kaplan-Meier survival curves were used to describe OS. There was no statistically significant difference in OS between patients with AL-PCMM and those with AL-MM (Fig. S1; P=0.354). In AL-MM and AL-PCMM, the survival time was significantly shorter in patients with an ALP ≥ 187.5 IU/L (Fig. 1A; median survival time of 1.0 vs. 32.0 months; *P*<0.001), GGT ≥ 85 IU/L (Fig. 1B; median survival time of 4.0 vs. 30.0 months; *P*=0.008), TBIL ≥20 µmol/L (Fig. 1C; median survival time of 1.0 vs. 30.0 months; *P*=0.010), CTNI ≥ 0.1 ng/mL (Fig. 1D; median survival time of 1.0 vs. 30.0 months; *P*=0.010), EF < 50% (Fig. 1E; median survival time of 7.0 vs. 30.0 months; *P*=0.015), and BU staging system stage ≥ III (Fig. 1F; median survival time of 1.0 vs. 30.0 months; *P*=0.002). Subsequently, we analyzed the survival times of patients with AL-MM and AL-PCMM by affected organ. The results showed that the median survival time was significantly shorter in patients with heart involvement (Fig. 1G; median survival time of 11.0 vs 58.0 months; *P*=0.038) and liver involvement (Fig. 1H; 5.0 vs 30.0 months; *P*=0.026). Interestingly, the median survival of patients with both liver and heart involvement was the same as that of patients with heart involvement only (Fig. 1I; 25.0 vs 58.0 months; *P*=0.114).

### Univariate and multivariate COX regression analyses of patients with AL-PCMM and AL-MM

Univariate Cox regression analyses found that ALP ≥ 187.5 IU/L (*P*<0.001; HR=6.694), GGT ≥ 85 IU/L (*P*=0.013; HR=2.767), EF < 50% (*P*=0.023; HR=2.809), TBIL ≥ 20 µmol/L (*P*=0.019; HR=3.698), CTNI ≥ 0.1 ng/mL (*P*=0.016; HR=2.792), ITE < VGPR (*P*=0.002; HR=6.711), and BU staging system stage ≥ III (*P*=0.004; HR=3.431) were related to the prognosis of AL-PCMM and AL-MM. Sex, age, Hb, bone destruction, Ca, creatinine, 24-h UTP, albumin, BNP, LDH, β2-MG, treatment regimen, and bone marrow plasma cell ratio did not affect the prognoses of AL-PCMM and AL-MM. However, age, Alb, ALP, GGT, TBIL, BNP, CTNI, BU staging system, and initial therapeutic effect were included in multivariate Cox regression analysis. Multivariate Cox regression analysis showed that a BU staging system stage ≥ III (*P*=0.001, HR=5.579), ALP ≥ 187.5 IU/L (*P*=0.011, HR=3.563), and ITE < VGPR (*P*=0.002, HR=7.462) were independent significant risk factors for poor prognosis (Table 3).

### Correlation of ALP with BNP, CTNI, and TBIL

The Kolmogorov-Smirnov test showed that ALP, CTNI, BNP, EF, and TBIL values were not normally distributed. To investigate the relationship between ALP and BNP, CTNI, EF, and TBIL levels, we performed Spearman's correlation tests. ALP at diagnosis was correlated with BNP (r=0.388;* P*=0.006; Fig. S2A), CTNI (r=0.313; *P*=0.028; Fig. S2B), and EF (r=-0.379; *P*=0.007; Fig. S2C). However, there was no correlation between ALP and TBIL levels (r=0.141; *P*=0.335; Fig. S2D).

## Discussion

Patients with AL-PCMM have a poorer prognosis than those with AL-only, similar to patients with AL-MM 16. We also found no statistically significant difference in OS between patients with AL-PCMM and those with AL-MM. However, why was there no difference in survival between these two groups of patients? We found that the indicators of tumor burden, such as Hb, bone destruction, Ca, Scr, β2-MG, LDH, and BMPC ratio, were not associated with the prognosis of AL-PCMM or AL-MM. However, amyloid organ involvement significantly impacts the prognosis of this group of patients, particularly those with cardiac amyloidosis. Compared with AL-PCMM, AL-MM displayed a difference in only the incidence of CRAB and BMPC ratio, which represented the tumor burden; however, there was no difference in the number or severity of involved organs, which resulted in death and a shorter survival time. Moreover, in our study, most patients were treated with the BD regimen, which resulted in no difference in treatment effect between these two groups of patients, resulting in this phenomenon. It should be noted that if these two groups of patients were treated with the recommended BCD or VRD regimen, the doses of cyclophosphamide and lenalidomide for those with AL-MM required adjustment according to the level of renal function, which might have led to a difference in the treatment effect and survival time of the two groups. However, this requires further investigation.

The Boston staging system, equivalent to the 2004 Mayo Clinical AL amyloidosis staging system, was used to predict the surival time in patients with AL amyloidosis, with a median survival time not reached for stage I, 30 months for stage II, and 5 months for stage III 3,4. Our study also demonstrated that the survival time was significantly shorter in patients with BU staging system stages III and IIIB than in those with BU staging system stages I and II (1 month vs. 30 months). In a previous study, the 2004 Mayo Clinical AL amyloidosis staging system and the 2012 Mayo Clinical AL amyloidosis staging system were independent poor prognostic factors for AL-PCMM and AL-MM 16. In our study, multivariate Cox regression analysis also found that a BU staging system stage ≥ III was an independent significant risk factor for a poor prognosis in AL-PCMM and AL-MM. The clinical stage of cardiac amyloidosis is the main factor affecting the prognosis of such patients. Therefore, more attention should be paid to evaluating cardiac amyloidosis severity in clinical settings. In addition, the BU staging system will be particularly widely used in this group of patients because not all centers have the capability to test NT-proBNP levels. However, whether the predictive value of the BU and Mayo staging systems is equivalent in this group of patients requires external validation.

Studies have reported that total bilirubin, an indicator of liver involvement severity in AL amyloidosis, is an independent adverse risk factor for progression-free survival (PFS) and OS in AL amyloidosis 19,20. However, in our study, multivariate Cox regression analysis showed that TBIL level was not an independent prognostic factor for AL-PCMM and AL-MM. Therefore, liver involvement appears to have little effect on the survival time of these patients. Interestingly, we found that an ALP ≥ 187.5 IU/L, which was the diagnostic criterion for liver involvement, was an independent prognostic factor for AL-PCMM and AL-MM. In a retrospective study, Raymond et al. found that an increased ALP level was associated with increased 1-year mortality 20. However, whether ALP is related to liver involvement severity and, thus, affects the survival time of these patients remains unclear. We found that ALP was related to BNP, CTNI, and EF rather than TBIL, which suggests liver involvement severity.

There may be two reasons for the elevated ALP levels in patients with primary systemic AL amyloidosis. First, chronic heart failure caused by cardiac amyloidosis leads to liver congestion, which manifests as elevated levels of biliary-related enzymes. Some studies also found that ALP was related to central venous pressure (CVP) and right ventricular free wall strain in patients with chronic heart failure, which supports this finding 21. Second, amyloid deposition in the liver obstructs the small bile ducts, leading to increased ALP levels. In our study, the former may have been even stronger. Therefore, ALP, which is related to cardiac amyloidosis rather than liver involvement, can be used as a prognostic factor for this group of patients. Moreover, as ALP is closely related to right heart failure caused by cardiac amyloidosis, ALP ≥ 187.5 IU/L may have a high misdiagnosis rate as a diagnostic criterion for liver involvement. Liver puncture can be actively improved to assess the probability of misdiagnosis when the patient has cardiac amyloidosis with an ALP ≥ 187.5 IU/L under safe conditions. In addition, it may be necessary to identify more suitable specific indicators to diagnose liver involvement.

Our study had some limitations. We lacked data from the 2004 and 2012 Mayo Clinic AL amyloidosis staging systems; thus, we could not determine whether the Mayo staging system was a predictor of a poor prognosis for this type of patient. In addition, we had insufficient data to evaluate the degree of hematological remission and organ response to AL amyloidosis in each patient. Therefore, we could not determine whether the 2016 International Myeloma Working Group consensus criteria for the response to multiple myeloma are correlated with the hematologic response criteria for AL amyloidosis; in principle, we should not include patients whose treatment effects cannot be evaluated. However, because the number of cases was too small, we classified the treatment effect of these patients as disease progression to ensure that a sufficient number of cases were included in the group. This may have resulted in a certain degree of bias.

## Conclusions

In summary, our study found that ALP, which is related to cardiac amyloidosis rather than liver involvement, can be a prognostic factor for this group of patients. BU staging system stage ≥ III, ALP ≥ 187.5 IU/L, and ITE < VGPR representing the degree of AL amyloidosis rather than tumor burden of multiple myeloma are independent significant risk factors for a poor prognosis of AL-PCMM and AL-MM.

## Supplementary Material

Supplementary figures and tables.

## Figures and Tables

**Table 1:** Clinical characteristics of AL-PCMM and AL-MM

	Amyloidosis n=49, cases (%)	AL-MM n=28, cases (%)	AL-PCMM n=21, cases (%)	P
Age ≥ 65 years	14 (28.5)	6 (21.4)	8 (30.1)	0.222
Male sex	34 (69.3)	18 (64.2)	16 (76.1)	0.533
Alb < 25 g/L	20 (40.8)	11 (39.2)	9 (42.8)	1.000
24-h UTP > 3.5 g	23 (46.9)	10 (35.7)	13 (61.9)	0.088
ALP ≥ 187.5 IU/L	7 (14.2)	4 (14.2)	3 (14.2)	1.000
GGT ≥ 85 IU/L	11 (22.4)	6 (21.4)	5 (23.8)	1.000
TBIL ≥ 20 µmol/L	5 (10.2)	3 (10.7)	2 (9.5)	1.000
CTNI ≥ 0.1 ng/mL	11 (22.4)	7 (25.0)	4 (19.0)	0.737
BNP ≥ 1500 pg/mL	7 (14.2)	4 (14.2)	3 (14.2)	1.000
EF < 50%	9 (16.3)	6 (21.4)	3 (14.2)	0.714
BU staging system stage ≥ III	10 (20.4)	7 (25.0)	3 (14.2)	0.482
LDH ≥ 240 IU/L	13 (26.5)	9 (32.1)	4 (19.0)	0.348
β2-MG > 3.5 mmol/L	38 (77.5)	23 (82.1)	15 (71.4)	0.494
BMPC ratio ≥ 20%	21 (34.6)	16 (57.1)	5 (23.8)	**0.024**
ITE < VGPR	30 (61.2)	16 (57.1)	14 (66.6)	0.564

24-h UTP, 24-h urine total protein; Alb, albumin; AL-MM, amyloid light-chain with multiple myeloma; ALP, alkaline phosphatase; AL-PCMM, amyloid light-chain and a bone marrow plasma cell ratio > 10%; β2-MG, β-2 microglobulin; BMPC, bone marrow plasma cell; BNP, brain natriuretic peptide; BU, Boston University; CTNI, cardiac troponin I; EF, ejection fraction; GGT, γ-glutamyl transpeptidase; ITE, initial therapeutic effect; LDH, lactate dehydrogenase; TBIL, total bilirubin

**Table 2:** Treatment of patients with AL-PCMM versus AL-MM

Treatment		AL amyloidosis n=49	AL-PCMM n=21	AL-MM n=28
Two-drug	BD	33	13	20
	MP	3	2	1
	RD	1	1	0
Three-drug	PAD	2	1	1
	RVD	2	1	1
	MPT	1	0	1
	BCD	1	1	0
	BTD	1	0	1
No treatment		5	2	3

AL, amyloid light-chain; AL-MM, AL amyloidosis with multiple myeloma; AL-PCMM, AL amyloidosis and a bone marrow plasma cell ratio > 10%; BCD, bortezomib plus cyclophosphamide and dexamethasone; BD, bortezomib and dexamethasone; BTD, bortezomib plus thalidomide and dexamethasone; MP, melphalan and prednisone; MPT, melphalan plus prednisone and thalidomide; PAD, bortezomib plus doxorubicin and dexamethasone; RD, lenalidomide and dexamethasone; RVD, bortezomib plus lenalidomide and dexamethasone.

**Table 3:** Cox regression analyses of 49 patients with AL-PCMM and AL-MM

Variable	Univariate		Multivariate	
	OS HR (95% CI)	P value	OS HR (95% CI)	P value
ITE < VGPR	6.711 (2.020-22.22)	0.002	7.462 (2.083-27.027)	0.002
ALP ≥ 187.5 IU/L	6.694 (2.609-17.174)	<0.001	3.563 (1.343-9.453)	0.011
BU staging system stage ≥ III	3.431 (1.477-7.969)	0.004	5.579 (2.018-15.420)	0.001
CTNI ≥ 0.1 ng/mL	2.792 (1.208-6.452)	0.016		
EF < 50%	2.809 (1.153-6.842)	0.023		
GGT ≥ 85 IU/L	2.767 (1.237-6.187)	0.013		
TBIL ≥ 20 µmol/L	3.698 (1.242-11.015)	0.019		
BNP ≥ 700 pg/mL	1.725 (0.836-3.560)	0.140		
Male sex	1.388 (0.901-2.138)	0.137		
Age ≥ 65 years	1.985 (0.917-4.298)	0.082		
Alb < 25 g/L	1.405 (0.679-2.906)	0.359		
Hb > 85 g/L	0.275 (0.065-1.172)	0.081		
Bone destruction > 3	0.828 (0.397-1.726)	0.615		
Ca > 2.65 mmol/L	0.733 (0.098-5.496)	0.763		
With MM	0.686 (0.323-1.457)	0.327		
24-h UTP > 3.5 g	1.120 (0.537-2.337)	0.762		
β2-MG > 3.5 mmol/L	1.130 (0.490-2.604)	0.774		
Scr ≥ 177 µmol/L	0.467 (0.162-1.349)	0.159		
LDH ≥ 240 IU/L	1.033 (0.456-2.340)	0.937		
BMPC ratio ≥ 20%	1.070 (0.513-2.231)	0.858		
Treatment regimen	0.285 (0.039-2.108)	0.219		

24-h UTP, 24-h urinary protein quantity; β2-MG, β-2 microglobulin; Alb, albumin; ALP, alkaline phosphatase; BMPC, bone marrow plasma cell; BNP, brain natriuretic peptide; BU, Boston University; CI, confidence interval; CTNI, cardiac troponin I; EF, ejection fraction; GGT, γ-glutamyl transpeptidase; Hb, hemoglobin; HR, hazard ratio; ITE, initial therapeutic effect; LDH, lactate dehydrogenase; MM, multiple myeloma; OS, overall survival; Scr, serum creatinine; TBIL, total bilirubin; VGPR, very good partial response
