# Supplementary material for: Prognostic Factors of AL-PCMM and AL-MM: A Single-Center Retrospective Study
Source: Int J Med Sci. 2022 Mar 14;19(3):588–95. doi: 10.7150/ijms.61712 (PMC8964324; doi:10.7150/ijms.61712)
Supplement: Supplementary file 1 — Supplementary figures and tables. [file ijmsv19p0588s1.pdf]

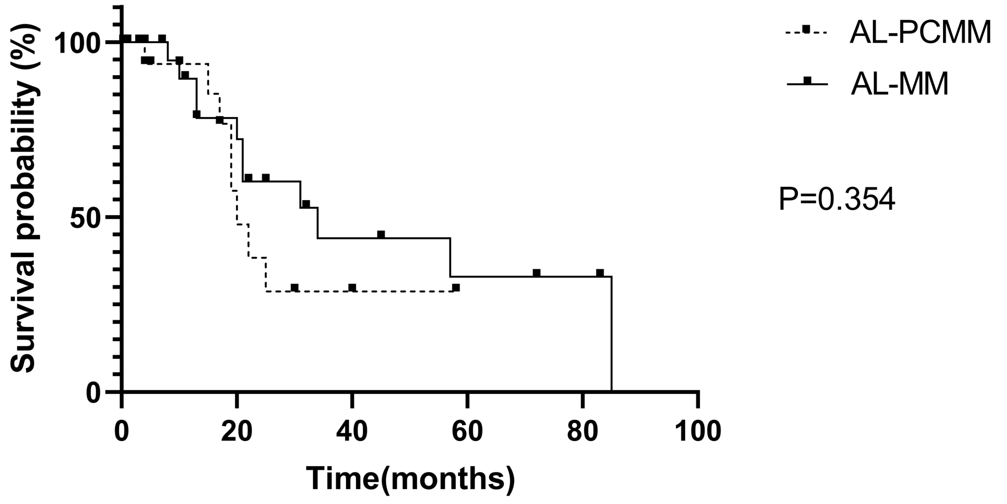

Figure S1: Kaplan–Meier survival curves of AL-PCMM and AL-MM: There was no statistically significant difference in overall survival between patients with AL-PCMM and AL-MM (*P*=0.354). AL-MM, amyloid light-chain with multiple myeloma; AL-PCMM, amyloid light-chain and a bone marrow plasma cell ratio > 10%.

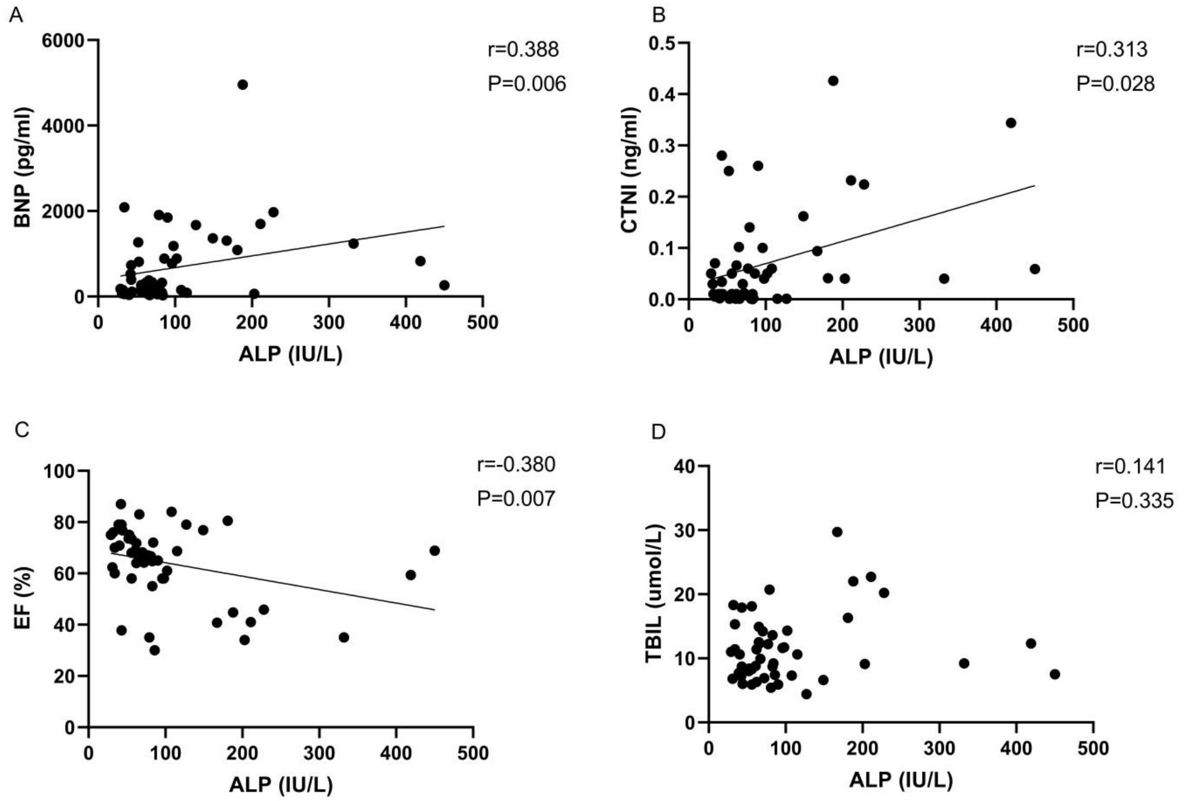

Figure S2: The Correlation between ALP and markers of cardiac amyloidosis and liver involvement: A. The positive correlation between BNP and ALP (r=0.388; *P*=0.006). B. The positive correlation between CTNI and ALP (r=0.313; *P*=0.028). C. The positive correlation between EF and ALP (r=-0.380; *P*=0.007). D. No correlation between TBIL and ALP (r=0.141; *P*=0.335). ALP, alkaline phosphatase; BNP, brain natriuretic peptide; CTNI, cardiac troponin I; EF, ejection fraction; TBIL, total bilirubin.

Table S1: Organ involvement in patients with AL-PCMM and AL-MM

Organ involvement	AL amyloidosis N=49	AL-MM N=28	AL-PCMM N=21	*P*
Kidney	36（73.4%）	20（71.4%）	16（76.1%）	0.708
Heart	27（55.1%）	17（60.7%）	10（47.6%）	0.361
Liver	13（26.5%）	7（25.0%）	6（28.5%）	0.779
Skin	8（16.3%）	4（14.3%）	4（19%）	0.655
Gastrointestinal tract	6（12.2%）	5（17.8%）	1（4.7%）	0.219
Soft tissue	4（8.2%）	2（7.1%）	2（9.5%）	1.000
Nerve	4（8.2%）	2（7.1%）	2（9.5%）	1.000
lung	1（2%）	1（3.6%）	0（0%）	1.000
< three ograns	34（69.4%）	18（64.2%）	16（76.2%）	0.371
≥ three organs	15（30.6%）	10（35.8%）	5（23.8%）	0.371

Table S2: Causes of death in all patients with AL-PCMM and AL-MM

Cause of death	Number of deaths	*Proportion
Heart	13	41.9%
Gastrointestinal tract	2	6.5%
Kidney	2	6.5%
Other deaths	2	6.5%
Unknown causes of death	12	38.6%

*Proportion: proportion of known causes of death; Heart, Cardiac amyloidosis; Gastrointestinal tract, Gastrointestinal involvement; Kidney, renal involvement; Other deaths, infection and hypercalcemia.
